# MRI and FDG PET/CT Findings for Borderline Brenner Tumor of the Ovary: A Case Report and Literature Review

**DOI:** 10.1155/2020/8878649

**Published:** 2020-08-17

**Authors:** Hiroki Matsutani, Go Nakai, Takashi Yamada, Kazuhiro Yamamoto, Masahide Ohmichi, Keigo Osuga

**Affiliations:** ^1^Department of Diagnostic Radiology, Osaka Medical College, 2-7 Daigaku-machi, Takatsuki, Osaka 569-8686, Japan; ^2^Department of Pathology, Osaka Medical College, 2-7 Daigaku-machi, Takatsuki, Osaka 569-8686, Japan; ^3^Department of Obstetrics and Gynecology, Osaka Medical College, 2-7 Daigaku-machi, Takatsuki, Osaka 569-8686, Japan

## Abstract

The imaging features of borderline Brenner tumor (BT) of the ovary are very limited, especially regarding apparent diffusion coefficient (ADC) value and 18F-fluorodeoxyglucose positron emission tomography (FDG-PET)/CT. We report a case of borderline BT in a 54-year-old woman with diffusion-weighted imaging (DWI) and FDG-PET/CT findings. Furthermore, ADC values and maximum standardized uptake value (SUV max) in the present case were compared with those of an additional 7 cases of benign BT in this institution in addition to literature reviews. Magnetic resonance imaging (MRI) revealed a pelvic unilocular cystic tumor with two solid components. The solid mass showing a low signal intensity (SI) in T2-weighted images (T2WI) and DWI was diagnosed as a benign BT histologically. The papillary tumor adjacent to the solid mass showing intermediate SI in T2WIs and high SI on DWI was a borderline BT. The mean ADC value (×10^−3^ mm^2^/s) of benign BTs (*n* = 7) and benign component in this case (*n* = 1) was 1.13, and the range of ADC values was broad (0.51–1.8). While, the ADC value of borderline Brenner component in this case was 1.10. The mean SUVmax of the benign BTs (*n* = 4) demonstrated mild FDG uptakes (2.3, range 1.9–2.6) in contrast with moderate FDG uptake (SUVmax: 5.8) of borderline Brenner component in this case and high FDG uptake (SUVmax: 9.6) of a malignant BT in a previous report. ADC values for the solid component of BTs are not useful for differentiating benign from malignant or borderline components, whereas PET/CT could be useful.

## 1. Introduction

Brenner tumors, which are rare and account for approximately 2% of all ovarian tumors [1], were identified in 1907 by Fritz Brenner [2]. The benign Brenner tumors are the most common, representing about 95% of the total, while the borderline tumors represent about 5% and the malignant ones less than 1% [3]. There have been several reports that describe the imaging features of benign Brenner tumors, although only a few relating to borderline and malignant Brenner tumors.

There have been only two reports describing magnetic resonance imaging (MRI) findings for borderline Brenner tumors [4, 5], and no reports have yet appeared relating to the use of diffusion-weighted imaging (DWI) and apparent diffusion coefficient (ADC) values in these cases. In addition, no findings concerning the use of positron emission tomography with fluorine-18 fluorodeoxyglucose (FDG-PET) of ovarian borderline Brenner tumor have yet been reported.

We report here a case of borderline Brenner tumor with solid components showing slight hyperintensity on DWI and moderate FDG uptake. Furthermore, the ADC and FDG uptake values for this case were compared with those from 7 additional cases of benign Brenner tumor from operations in our institutions as well as those reported in previous reports.

## 2. Case Presentation

A 54-year-old, gravida 4, para 2 woman with clinical symptoms of abdominal distension was referred to our hospital because of a pelvic mass detected by ultrasonography at another hospital. Transvaginal ultrasonography revealed a 118 × 85 mm unilocular cystic mass with a papillary mural mass in the pelvis, suggesting ovarian cancer. However, the serum levels for cancer antigen 125 (CA125), carcinoembryonic antigen (CEA), and carbohydrate antigen 19-9 (CA19-9) were all within normal limits. Blood cell counts and blood biochemistry were normal.

**Figure 1:**
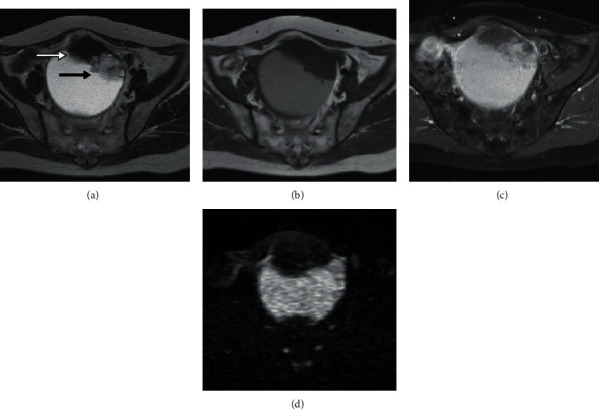
MR findings for the borderline Brenner tumor in a 54-year-old woman. (a) The axial T2WI shows a unilocular cystic tumor in the pelvic cavity which was 9 cm in diameter with two solid components. The cystic content has the same signal intensity (SI) as water. The solid mass located at the anterior wall (white arrow) shows low SI similar to the SI of muscle. The papillary tumor on the left side of the solid mass (black arrow) shows an intermediate SI, slightly higher than the SI of muscle. (b) The axial T1WI shows the cystic content has slightly higher SI than water. The solid mass shows slightly lower SI than muscle. The papillary tumor on the left side of the solid mass shows low SI, similar to the SI for muscle. (c) Gadolinium-enhanced fat-saturated T1WI shows the solid mass is slightly enhanced and the papillary tumor is well enhanced. (d) Axial DWI (*b* = 0, 1000 s/mm^2^) shows a solid mass with a low SI and the papillary tumor with high SI.

MRI revealed a unilocular cystic tumor which was 9 cm in diameter with two solid components in the pelvic cavity. The cystic content had slightly higher signal intensity (SI) than water in T1-weighted images (T1WIs), and the same SI as water in T2-weighted images (T2WIs). The solid mass located at the anterior wall showed a low SI similar to the SI of muscle in T2WIs and T1WIs, slightly enhanced on gadolinium-enhanced, fat-saturated T1WIs, and a low SI on DWI (Figure 1). The adjacent papillary tumor on the left side of the solid mass showed an intermediate SI, slightly higher SI than muscle in the T2WIs, well enhanced on contrast-enhanced fat-saturated T1WIs, and a high SI on DWI (Figure 1). The ADC value for the solid mass obtained from DWI (*b* = 0, 1000 s/mm^2^) was 0.51 × 10^−3^ mm^2^/s, whereas that for the papillary tumor was 1.10 × 10^−3^ mm^2^/s.

**Figure 2:**
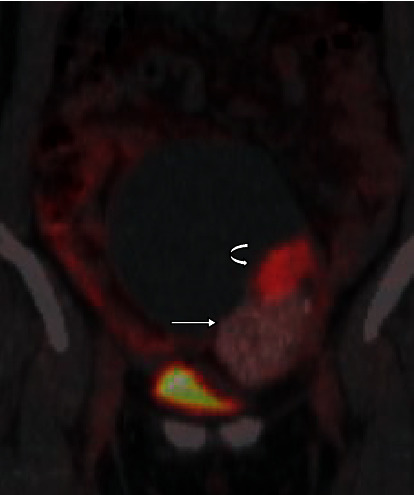
Coronal fused PET/CT image of the borderline Brenner tumor. The PET/CT shows a mild FDG uptake (SUVmax: 2.3) and calcification in the solid component (arrow), and moderate FDG uptake (SUVmax: 5.8) in the papillary tumor (curved arrow).

Positron emission tomography/computed tomography (PET/CT) findings revealed mild FDG uptake (SUVmax: 2.3) and calcification in the solid mass, and moderate FDG uptake (SUVmax: 5.8) in the papillary tumor (Figure 2). Lymph node metastasis and distant metastasis were not detected.

**Figure 3:**
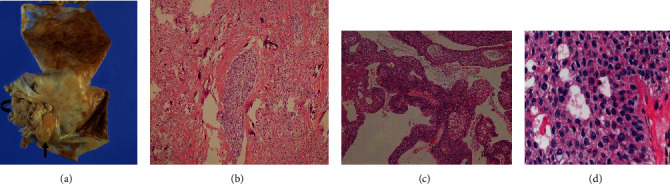
Pathology findings for the borderline Brenner tumor. (a) Macroscopically, the solid mass is hard and yellow-tan (arrow) in color and is accompanied by the papillary tumor (curved arrow). (b) A low-power microscopic examination of the solid mass with a low signal intensity in T2WIs demonstrates solid islands of epithelial cell nests in the fibrous stroma with hyalinization and calcification, indicating a benign Brenner tumor. (c) A low-power microscopic examination of the papillary tumor shows mucinous columnar epithelium and transitional epithelial cells without invasion. (d) A higher power image of the papillary tumor shows transitional epithelial cells that have longitudinal grooving with mild nuclear atypia, indicating a borderline Brenner tumor.

Gynecologists performed surgeries including abdominal hysterectomy, bilateral salpingo-oophorectomy, pelvic and para-aortic lymph node dissection, and omentectomy. At surgery, the smooth, whitish tumor without adhesion to surrounding tissue originated from the left ovary and measured 12 cm in diameter. A small amount of ascites was present in the pelvis, and peritoneal washing cytology gave a false positive during the surgery. Macroscopically, the cystic content was dark brownish, and a smooth solid mural mass accompanied by partial papillary projection was present (Figure 3(a)).

Subsequently, transabdominal abdominal hysterectomy with bilateral salpingo-oophorectomy, omentectomy, and pelvic and para-aortic lymphadenectomy were performed. Histological examination revealed that the solid component with low SI in the T2WIs showed solid islands of epithelial cell nests and longitudinal grooving in fibrous stroma with hyalinization and calcification, indicating a benign Brenner tumor (Figure 3(b)). On the other hand, the partial papillary projection showed mucinous columnar epithelium and transitional epithelial cells with mild nuclear atypia without invasion (Figure 3(c)–3(d)). Thus, the final histological diagnosis was borderline Brenner tumor of the ovary. Clinical and radiologic assessment over a 30-month follow-up has shown no evidence of tumor recurrence.

## 3. Review of the Literature

We searched the PubMed database for previous cases published in English from 1956 to 2019 using the keyword terms “Brenner”, “ovary”, and “imaging”.

Only one report was found describing the ADC value for a malignant Brenner tumor including that of a benign Brenner component [6]. We retrospectively reviewed apparent ADC values from eight cases of benign Brenner tumor proven from pathology including a benign Brenner component in the present case in our institutions from July 2006 to December 2017. All ADC values for these cases were obtained by using 1.5-T superconducting units. Although the mean ADC value for the 8 cases was 1.13 × 10^−3^ mm^2^/s, the range of ADC values was broad (0.51–1.8 × 10^−3^ mm^2^/s). On the other hand, the ADC value for the borderline Brenner component in the present case was 1.10 × 10^−3^ mm^2^/s. The ADC value for the malignant Brenner tumor reported by Kikukawa et al. [6] was 0.84 × 10^−3^ mm^2^/s.

Only two reports were found concerning PET/CT imaging for Brenner tumors. FDG PET/CT scans were obtained in four patients including the present case in our institution. All tumors for the four patients with a benign Brenner tumor, including the benign Brenner component in the present case, demonstrated mild FDG uptakes (mean SUVmax: 2.3, range 1.9–2.6). The single report describing the SUVmax for a benign Brenner tumor showed a mild FDG uptake (SUVmax: 1.9) [7], consistent with our findings. On the other hand, the borderline Brenner component in the present case showed moderate FDG uptake (SUVmax: 5.8), and a malignant Brenner tumor from a previous report showed a high FDG uptake (SUVmax: 9.6) [8].

A summary of the ADC values and maximum standardized uptake values for benign, borderline, and malignant Brenner tumors measured in our institution as well as in previous reports is shown in Table 1.

## 4. Discussion

Brenner tumors are an uncommon subtype of the epithelial tumors of the ovary and are classified into three categories: benign, borderline, and malignant according to the World Health Organization (WHO) classification, revised in 2014. A pathologically benign Brenner tumor consists of nests of bland, transitional-type cells within an abundant fibromatous stroma. Although Brenner tumors are commonly solid, they are sometimes associated with mucinous tumors with cystic changes because they can exhibit marked mucinous metaplasia [9]. Nucleoli of the transitional-type cells typically contain longitudinal grooves. Focal or extensive calcification is often observed due to prominent hyalinization of the fibromatous stroma. Benign Brenner tumors show a homogenous low SI in T2WIs and show patchy enhancement on gadolinium-enhanced T1WIs, reflecting its abundant fibrous stroma [10]. On the other hand, borderline Brenner tumors are presumed to arise from benign Brenner tumors; therefore, a solid area of benign Brenner tumor is nearly always present in the pathology. They typically show a large cystic mass with a mural papillary projection into the cystic lumen. In terms of the pathology, the papillary projection consists of atypical proliferative cells resembling low-grade, noninvasive papillary transitional cell tumors.

To our knowledge, only two reports describing MRI findings for a borderline Brenner tumor [4, 5]. One case report demonstrated a cystic mass with thickened septa and a papillary projection showing relatively low SI higher than the SI for muscle in T2WIs, and rapid enhancement followed by persistent enhancement in dynamic contrast-enhanced T1-weighted fat-suppressed images according to a case report [4]. However, the other study reported different findings such as multilocular cystic masses with thickened septa showing an iso-SI compared with that of adjacent muscle in T2WIs and a lower SI compared with that of myometrium in contrast-enhanced T1WIs [5]. The difference in the SI of the solid component in T2WIs between a benign and borderline Brenner tumor may be related to less intervening fibrous stroma in a borderline Brenner tumor than in a benign Brenner tumor.

In the present case, the mural solid mass was divided into two components in terms of the morphology and the SI. One component showed a smooth surface with low SI in the T2WIs, DWI, and ADC map; the other showed a papillary shape with an intermediate SI in the T2WIs and high SI on the DWI and ADC map. From pathology, they corresponded to a benign and borderline Brenner tumor, respectively. Considering the cellular density, the benign Brenner component is likely to have higher ADC value than the borderline Brenner component, but our result was contrary. According to the ADC values from our nine benign Brenner tumors which took a broad value (ADC value: 0.51 − 1.8 × 10^−3^ mm^2^/s), the measured ADC values were not likely to be useful for differentiating benign from malignant or borderline Brenner tumor. There are two reasons why benign Brenner tumors take a broad range of ADC values. First, a T2 blackout effect might affect the ADC value for a benign Brenner tumor because the tumor tends to show low SI in the T2WIs. Second, the ADC value for a benign Brenner tumor can be influenced by the location of the region of interest (ROI) on the tumor because of histological complexities including solid islands of epithelial cell nests, and fibrous stroma with or without calcification (Figure 3(b)). Mukuda also reported that the variation of the shapes of the ROI influences the ADC values in ovarian tumors [11]. On the other hand, Kikukawa et al. [6] reported that DWI and ADC values may be useful in distinguishing malignant from benign solid components in malignant Brenner tumors and may be a diagnostic clue to this rare tumor; although, only a single case report described the DWI and ADC values for a malignant Brenner tumor.

Regarding the FDG-PET findings for ovarian Brenner tumors, there have been reports of the use of FDG-PET for ovarian malignant Brenner tumors and benign Brenner tumors, one each [7, 8]. However, those for borderline Brenner tumors have never been reported. In our case, PET/CT could be useful in distinguishing a benign component from a borderline component using the relative difference in FDG uptake. In the previous English-language literature, there has been only one report describing FDG PET/CT findings for a benign Brenner tumor, and only one report describing FDG PET/CT findings for a borderline Brenner tumor. Toriihara et al. [7] reported that the solid component of a benign Brenner tumor demonstrated mild FDG uptake (SUVmax: 1.9) and contained calcification. On the other hand, Mena et al. [8] reported that in PET/CT the malignant Brenner tumor of the ovary showed a heterogeneous high FDG uptake (SUVmax: 9.6). Following these results that include our case of borderline Brenner tumor, the FDG uptake for the Brenner tumor is likely to correlate with the grade of malignancy. Moreover, there are some reports on the utility of FDG uptake for differentiating benign and borderline/malignant ovarian lesions. Kitajima et al. [12] showed that preoperative SUVmax was significantly different between benign and malignant lesions, and the sensitivity, specificity, and accuracy of PET/CT when using a cutoff SUVmax of 2.75 to separate malignant from borderline/benign lesions were 86.3, 73.7, and 82.0%, respectively. Tanizaki et al. also showed that an SUVmax cutoff value of 2.9 had a sensitivity of 80.6%, specificity of 94.6%, positive predictive value (PPV) of 91.5%, and negative predictive value (NPV) of 87.1% for detecting a malignancy [13]. Both reports are compatible with our four patients with benign Brenner tumors, including a benign Brenner component in the present case, demonstrating mild FDG uptake values (SUVmax range: 1.9-2.6). But further studies should be needed to clarify if the tumor FDG uptake correlates with its grade of malignancy in Brenner tumor.

We present here a case of borderline Brenner tumor which appeared as a cystic mass with a solid mass showing low SI in the T2WIs accompanied by a papillary tumor showing intermediate SI in the T2WIs and high SI on DWI. However, the ADC values for the solid component of Brenner tumors are not useful for differentiating benign from malignant or borderline component, while PET/CT could be useful in distinguishing benign from malignant or borderline solid components in Brenner tumors.

## Figures and Tables

**Table 1:** The summary of ADC values and maximum standardized uptake values of Brenner tumors measured in our institution in addition to previous reports.

No	Author (year)	Age (year)	Pathological findings	ADC (10^−3^ mm^2^/s)	SUVmax
1	Present case	49	Benign	1.5	N.A.
2	Present case	65	Benign	0.87	2.6
3	Present case	71	Benign	0.9	N.A.
4	Present case	68	Benign	1.9	N.A.
5	Present case	75	Benign	0.82	N.A.
6	Present case	45	Benign	1.8	1.9
7	Present case	64	Benign	0.77	2.4
8	Present case∗	54	Benign component	0.51	2.2
			Borderline component	1.1	5.8
9	Kikukawa (2012)	85	Benign component	1.15	N.A.
			Malignant component	0.84	N.A.
10	Toriihira (2012)	85	Benign	N.A.	1.9
11	Mena (2015)	59	Malignant	N.A.	9.6

^∗^Present case.

N.A.: not assessed.

## Abbreviations

ADC: Apparent diffusion coefficient
CA-125: Cancer antigen 125
CA19-9: Carbohydrate antigen 19-9
CEA: Carcinoembryonic antigen
DWI: Diffusion-weighted imaging
FDG: 2-deoxy-2-[fluorine-18] fluoro-D-glucose
MRI: Magnetic resonance imaging
PET/CT: Positron emission tomography with computed tomography
ROI: Region of interest
SI: Signal intensity
WI: Weighted imaging.
